# CircRNA screening and ceRNA network construction for milk fat metabolism in dairy cows

**DOI:** 10.3389/fvets.2022.995629

**Published:** 2022-11-10

**Authors:** Xiaofang Feng, Zhengyun Cai, Tong Mu, Baojun Yu, Ying Wang, Ruoshuang Ma, Jiaming Liu, Chuanchuan Wang, Juan Zhang, Yaling Gu

**Affiliations:** Ningxia Key Laboratory of Ruminant Molecular and Cellular Breeding, School of Agriculture, Ningxia University, Yinchuan, China

**Keywords:** dairy cows, circRNAs, milk fat percentage, ceRNAs, RNA-seq

## Abstract

**Background:**

Milk fat is one of the main reference elements for evaluating milk quality and is a primary objective trait in dairy cattle breeding. In recent years, circular RNAs (circRNAs) have been found to play crucial roles in many biological processes. However, the function and expression profiles of circRNAs in milk fat synthesis in cows are not completely understood. We performed RNA sequencing to analyze the genome-wide expression of circRNA transcripts in bovine mammary epithelial cells (BMECs) from cows with extreme differences in milk fat percentage. We identified candidate differential circRNAs associated with milk fat metabolism using functional enrichment analysis and constructed a lipid metabolism-related competing endogenous RNA (ceRNA) interactive regulatory network.

**Results:**

A total of 290 circRNAs were significantly differentially expressed (DE-circRNAs) in high milk fat percentage (HMF) cows compared to that in low milk fat percentage (LMF) cows. Of the 290 circRNAs, 142 were significantly upregulated and 148 were significantly downregulated. Enrichment analysis (Gene Ontology and Kyoto Encyclopedia of Genes and Genomes) identified four DE-circRNAs (circ_0001122, circ_0007367, circ_0018269, and circ_0015179) that potentially regulate milk fat metabolism. Among them, circ_0001122, circ_0007367, and circ_0015179 had relatively high expression levels in cow mammary gland tissue compared to other tissues (heart, liver, kidney, uterus, ovaries, and small intestine) of cows. The regulatory networks circ_0001122:miR-12043:LIPG, circ_0007367:miR-331-3p:CIDEA/PML, and circ_0018269:miR-11989:RORC/HPX are potential networks to explore the mechanism of milk fat regulation.

**Conclusions:**

These results reveal the possible role of circRNAs in milk fat metabolism in dairy cows. Several important circRNAs and ceRNAs affecting milk fat synthesis were identified, providing insights into the complex biology of milk fat synthesis as well as a novel theoretical perspective for future research on lactation, milk quality, and breed improvement in dairy cows.

## Background

Milk fat is not only a significant ingredient in the production of butter and yogurt but also an essential measure of milk quality and production performance, as well as vital for nutrient metabolism during human growth and development (1). Milk fat is an important source of essential fatty acids (2, 3), containing ~400 fatty acids, making it one of the most complex natural fats in terms of content and variety. Some medium- and short-chain saturated fatty acids from milk fat, such as C4:0, C8:0, C10:0, and C12:0, have been found to have anticancer, antiviral, and antibacterial properties, as well as the ability to delay tumor growth (4, 5). Oleic acid, the most abundant unsaturated fatty acid in milk, can reduce the levels of cholesterol, low-density lipoprotein cholesterol, and triglycerides (TAGs) in the blood plasma (6, 7). Similarly, conjugated linoleic acid and C18:2 have shown antioxidant effects against oxidative damage in cells caused by hydrogen peroxide (8).

Milk fat metabolism is a complex regulatory process involving *de novo* fatty acid synthesis, uptake and transport, TAG synthesis, and lipid droplet secretion (9). Although it has been intensely researched at the mRNA level, several non-coding RNAs are also known to regulate this specific molecular process (10–12). Among these, circRNAs have recently been identified as a class of RNAs that are widely present in mammals, acting as “miRNA sponges” by binding to proteins and inducing a variety of biological processes (13). Recent studies have found that some circRNAs can be translated into functional peptides or proteins that perform essential functions (14). In addition, studies have shown that circRNAs have significant regulatory effects on the growth and development of mammary epithelial cells and the regulation of milk fat metabolism. Zhang (15) found that circ0001186 could enhance bovine mammary epithelial cell viability and promote cell proliferation, acting as a miR-3432a “sponge” to competitively bind to miR-3432a, upregulating the expression of its target protein, JunD. Zhang et al. (10) demonstrated that circRNA-006258 could regulate TAG synthesis in sheep mammary epithelial cells by the adsorption of miR-574-5p. Similarly, Hao et al. (16) screened circRNA-001091 with differential expression in sheep mammary tissue and found that miR-432, miR-200b, and miR-29 were its target microRNAs, among which miR-29 has been found to regulate milk protein, TAG, and lactose secretion in BMECs (17).

However, at present, a comprehensive identification of valuable candidate circRNAs involved in milk fat metabolism is needed. To address this gap and identify more circRNAs related to milk fat metabolism, this study used RNA sequencing (RNA-seq) technology and bioinformatic methods to evaluate circRNA expression in BMECs from cows with different milk fat percentages. As a result, potential circRNAs for milk fat metabolism were screened, and a ceRNA interaction regulatory network was constructed. The resulting network provides a novel theoretical perspective for future research on milk fat metabolism in dairy cows and a basis for the molecular breeding of dairy cows.

## Materials and methods

### Milk sample collection and primary mammary epithelial cell isolation, culture, and characterization

Based on a whole year of dairy herd improvement determination (DHI) data from cows on the Ningxia Nongkeng Helanshan Maosheng dairy farm, we selected four cows with chronically high milk fat percentage and four cows with chronically low milk fat percentage from 245 primiparous cows. Cows were of similar daily milk yield (35.21–37.21 kg) and age (29–31 months old), and in the middle and late stages of lactation (150–220 days) and were fed and managed similarly (Supplementary Table 1; Table 1). Milk samples were collected from each cow three times a day (6 am, noon, and 6 pm) and mixed in a ratio of 4:3:3 for DHI determination to further identify eight non-sibling Holstein cows with extreme differences in milk fat percentage and somatic cell counts (SCC) <100,000/ml (Table 1). Fresh milk samples (200 ml) were aseptically collected into 50 ml centrifuge tubes, placed in a thermos flask containing 37°C sterile water, and returned to the laboratory and processed immediately.

**Table 1:** High and low milk fat percentage and somatic cell count of eight non-sibling Holstein cows.

**Items**	**ID**	**Age (month)**	**Lactation days**	**MFP (%)**	**DMY (kg)**	**SCC (10,000 per ml)**
HMF	H_2098	30	186	4.82	35.65	5
	H_2046	31	189	4.54	36.54	2
	H_2226	29	160	4.74	35.66	9
	H_2190	29	157	4.88	35.21	5
LMF	L_2034	31	187	2.60	36.74	6
	L_2037	31	175	2.81	35.56	5
	L_2170	30	207	2.85	36.75	8
	L_2137	29	150	2.84	37.21	7

HMF, high milk fat; LMF, low milk fat; ID, identification number of each cow; MFP, milk fat percentage; DMY, daily milk yield; SCC, somatic cell count, age: (in months) of each cow at the time of sampling.

Next, BMECs were isolated from the milk samples and cultured as follows: (1) The freshly collected milk samples were centrifuged (1,000 r/min for 20 min), and 5 ml of the turbid liquid at the bottom of the tube was retained. This mixture was then pipetted, mixed and transferred to a new sterile tube and an equal amount of phosphate-buffered saline (PBS) containing the penicillin-streptomycin-neomycin (PSN) antibiotic mixture (catalog no. PB180123, supplier: Procell, wuhan) was added. After mixing well, the tubes were centrifuged (1,000 r/min for 10 min) and the supernatant was removed. This procedure was repeated 3–5 times until the liquid at the bottom was clear. (2) The resulting liquid and precipitate were mixed by pipe tying up and down followed by addition in of an equal amount of PBS containing the PSN. This mixture was centrifuged (1,500 r/min for 10 min) and the supernatant was discarded. This step was repeated twice. (3) The pellet was resuspended in the complete medium containing the PSN, and then transferred to a T25 culture flask for cultivation. (4) The complete medium was replaced every other day, and the cells were washed with PBS containing PSN. Thereafter, the culture medium was changed every 3 days. The initial identification of the BMECs was based on their unique pebble shape, normal growth, and secretion characteristics. The isolated cells were then tested for keratin 18 (an epithelial cell-specific protein) by immunofluorescence; a positive result indicated that the isolated cells were epithelial cells (18).

### RNA-seq library construction and sequencing analysis

TRIzol reagent (Invitrogen, Thermo Fisher Scientific, USA) was used to extract the total RNA from BMECs of HMF and LMF cows and the RNA integrity was assessed (RNA Nano 6000 Assay Kit, Bioanalyzer 2100 system; Agilent Technologies, CA, USA). All samples had a 260/280 ratio between 1.70 and 1.90 and an RNA integrity index (RIN)≥8.00. RNA purity was verified using the NanoPhotometer^®^spectrophotometer (IMPLEN, CA, USA). Briefly, 5 μg of RNA per sample was used as the input material for the RNA sample preparations. Strand-specific libraries were constructed by removing ribosomal RNA using the Epicenter Ribozero™ rRNA Removal Kit (Epicenter, CA, USA). The rRNA-free residue was cleaned by ethanol precipitation. Then, the libraries were qualified and sequencing was performed on the Illumina PE150 platform.

The resulting raw data (raw reads) in fastq format were first processed through in-house Perl scripts to obtain clean data (clean reads) by removing adapter, ploy-N, and low-quality reads. At the same time, the Q20, Q30, and GC content of the clean data were calculated. When quality control was greater than Q20 and Q30 (false discovery rate <1% or 0.1%), the reference genome and gene model annotation files were downloaded from the genome website (https://bovinegenome.elsiklab.missouri.edu/downloads/ARS-UCD1.2). An index of the reference genome was built using bowtie 2 software (version 2.2.8) and the paired-end clean reads were aligned to the reference genome (19). Any reads that aligned to the reference genome more than 10 times were discarded. Then, transcripts per million (TPM) were calculated for RNA-seq analyses. The reads that were aligned to each repeat class were counted and the counts were normalized against the total number of aligned reads (whole genome) and the total length of each repeat class.

### Identification and expression level analysis of circRNAs

The circRNAs were detected and identified using find_circ (20) and CIRI2 (21). TPM was used to normalize known and novel circRNAs in each sample (22) as follows: normalized expression levels = (readCount × 1,000,000)/libsize, where libsize is the sum of circRNA read counts. The differential expression analysis of the transcript counts matrices of BMECs of HMF and LMF cows was performed using the “DESeq2” package in R (23). DESeq2 provides statistical routines for determining differential expression in digital gene expression data using a model based on the negative binomial distribution. The resulting *P*-values were adjusted using the Benjamini–Hochberg method for controlling the false discovery rate. Genes with an adjusted *P*-value were considered as differentially expressed (|log_2_ FoldChange|≥ 1, *P* < 0.05).

### Gene function classification and annotation

The “clusterProfiler” package in R (version 4.05) was used for the functional annotation of the genes. This package relies on the whole genome annotation package (OrgDb) project released by Bioconductor, which is updated semi-annually; that is, the gene functions annotated in this study are up to date (24). The enrichGO function was applied to the annotation of gene ontologies, including biological process (BP), molecular function (MF), and cellular component (CC), with the parameters set as follows: *pvalueCutoff* = 0.05 (adjusted *P*-value cutoff for enrichment test), *qvalueCutoff* = 0.2 (*q*-value cutoff for enrichment test), and *pAdjustMethod* = “BH” (multiple testing correction method for *P*-value, i.e., the Benjamini–Hochberg method). The maximum number of genes (maxGSSize) and the minimum number of genes (minGSSize) enriched in the pathway were adjusted according to the size of the annotated gene set. The enrichKEGG function was used for the annotation of the Kyoto Encyclopedia of Genes and Genomes (KEGG) to identify relevant signaling pathways, using the same parameters as in the enrichGO function. All enrichment analysis results were visualized using the “ggplot2” package in R.

### Target relationship prediction and ceRNA network construction

The target microRNAs (miRNAs) of the circRNAs, the target genes of the miRNAs, and their binding sites were predicted using TargetScan (version 7.2) and miRanda (version 3.3a) softwares. The present target prediction method is not a simple intersection of data but is truly integrated at the algorithmic level. miRanda is a dynamic programming algorithm based on RNA secondary structures and free energy (25) that can discover any seed-type sites, although the evaluation of the site sequences did not lend biological experimental data. TargetScan is based on the fitting of mRNA to miRNA expression profile data and identification of biologically meaningful site sequence characteristics and scoring models of relative conservation (26–28). However, its site-matching algorithm is rather naive and can only search for sites with a perfect match between nucleotides 2–7, such as m8, 7mer-m8, and 7mer-a1. By integrating the advantages of both algorithms, predictions can be made for arbitrary mRNA/circRNA targeting miRNA without missing any sites and without the constraints imposed by database versions and existing information, while ensuring that site metrics are biologically meaningful (29). Finally, the miRNAs and target genes in the ceRNA network were screened using Context+ and Free Energy as criteria. The resulting interrelationships between circRNAs/miRNAs/mRNAs were visualized using Cytoscape 3.8.2.

### Validation of circRNAs

TRIzol reagent was used to extract the total RNA from tissues of heart, liver, kidney, uterus, ovaries, small intestine, and mammary gland. Sequencing data results and circular structure validation using total RNA extracted from BMECs. According to the manufacturer's instructions, first-strand cDNA synthesis was performed using *PrimeScript RT* Reagent Kit with gDNA Eraser (Takara, Dalian, China). According to the manufacturer's instructions, quantitative reverse transcription PCR (RT-qPCR) was performed using SYBR Premix Ex Taq™II (TaKaRa) on a Bio-Rad CFX96 Touch™ Real-Time PCR Detection System (Bio-Rad, Hercules, CA, USA). The amplification procedure was as follows: initial denaturation at 95°C for 30 s, followed by 40 cycles of denaturation at 95°C for 5 s, and annealing for 30 s, 40 cycles. Divergent primers were designed using Primer Premier 5.0 and glyceraldehyde 3-phosphate dehydrogenase (*GAPDH*) was used as an internal reference. To verify the circular structure of the circRNAs, PCR amplification and Sanger sequencing were performed using divergent primers for circRNAs to verify their head-to-tail splicing. Supplementary Table 2 lists the primer sequences used in this study.

### Statistical analysis

Data were filtered using Microsoft Excel 2016, and the 2^−ΔΔCt^ method was used to analyze the relative expression of DE-circRNAs normalized to that of the *GAPDH* gene. Data are expressed as the mean ± standard deviation.

## Results

### Sequencing and characterization of circRNAs

**Figure 1:**
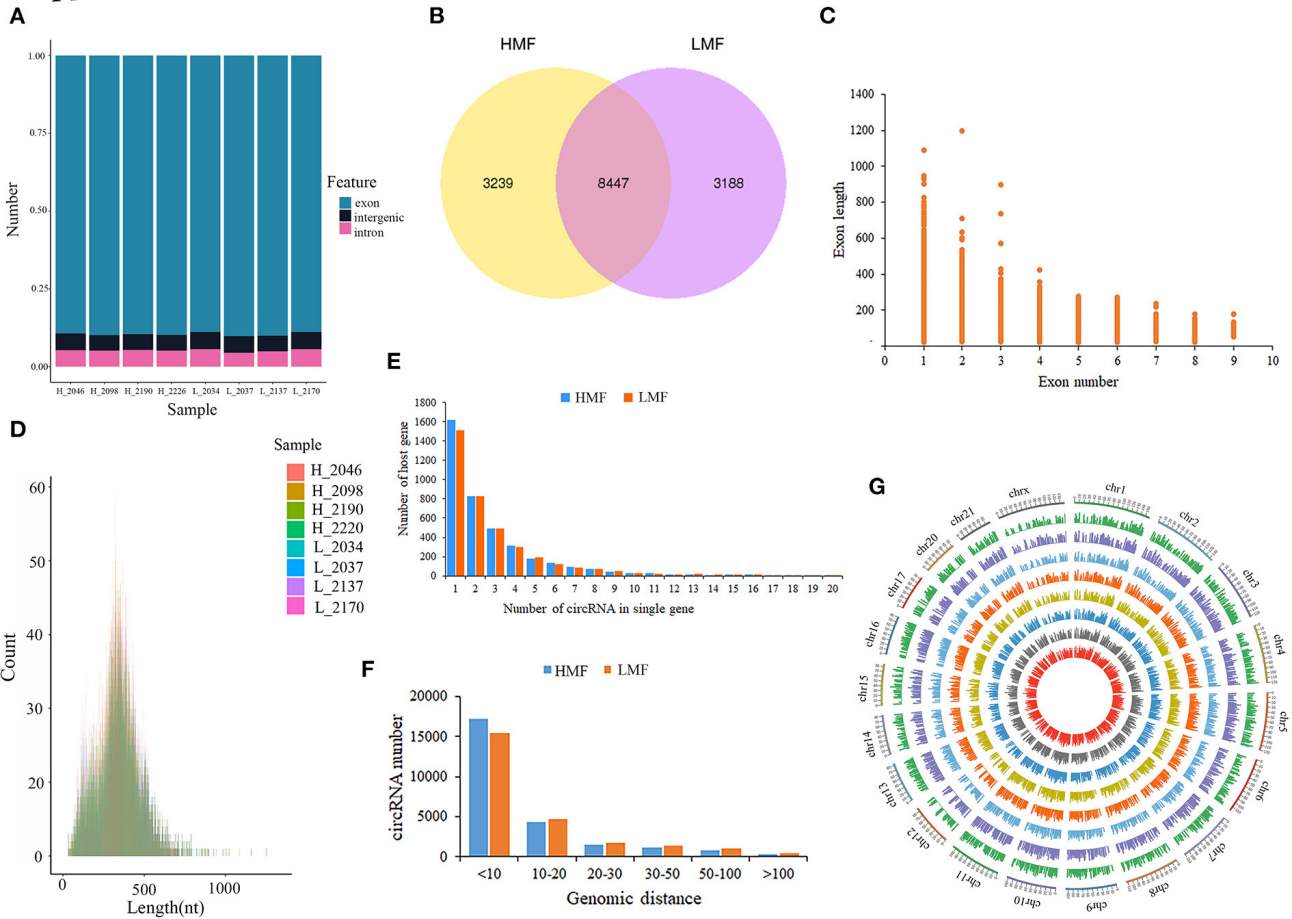
Characterization of circRNAs in mammary epithelial cells of cows. **(A)** Statistics of circRNAs sources. **(B)** Distribution of circRNAs in mammary epithelial cells of cows with high and low milk fat percentage. **(C,D)** Exon lengths of circRNAs. **(E)** Number of circRNAs produced by the same gene. **(F)** Genomic distance of circRNA reverse splice sites. **(G)** Chromosomal distribution of circRNAs.

The deep sequencing (sequencing depth: 10×, filtering criteria: depth < 3×) of BMECs from HMF and LMF cows yielded 62–70 million and 64–70 million clean reads, respectively. The Q20 of each sample was not <96.42%, and the Q30 was not <91.44% after quality control. The obtained effective reads were aligned to the bovine reference genome, with an average of 90.63% of reads able to align to the reference genome (Supplementary Table 3): 86.76% of circRNAs were from the exonic regions, 6.31% from the intergenic regions, and 7.02% from the intronic regions (Figure 1A). A total of 14,874 novel circRNAs were identified from the eight libraries (Supplementary Table 4): 3,239 circRNAs were expressed only in the HMF group, 3,188 circRNAs were expressed only in the LMF group, and 8,447 circRNAs were co-expressed in both groups (Figure 1B). The feature analysis of 14,874 circRNAs found that most circRNAs were composed of 1–4 exons, while a small portion consisted of 5–9 exons, with all exon lengths <1,400 nucleotides. In addition, the length of circRNAs from a single exon was longer than that of multiple exon circRNAs, and 81.09% of circRNAs (12,062) were within 500 nucleotides (Figures 1C,D). Although most circRNAs were produced by one host gene, there were also some host genes that generated 2–6 circRNAs, and a small portion of host genes made over 10 circRNAs (Figure 1E). Most circRNAs had back splice sites within 50 kb, and very few circRNAs had back splice sites longer than 100 kb, suggesting that circRNAs may be generated within the same gene region and have the potential to be developed throughout the process of RNA splicing (Figure 1F). circRNAs are widely distributed in bovine chromosomes; the longer the chromosome, the greater the number of splice sites, with the most significant number of circRNAs distributed at chrX, chr1, and chr2, followed by chr3–8 (Figure 1G).

### Differential expression analysis and functional annotation of circRNAs

**Figure 2:**
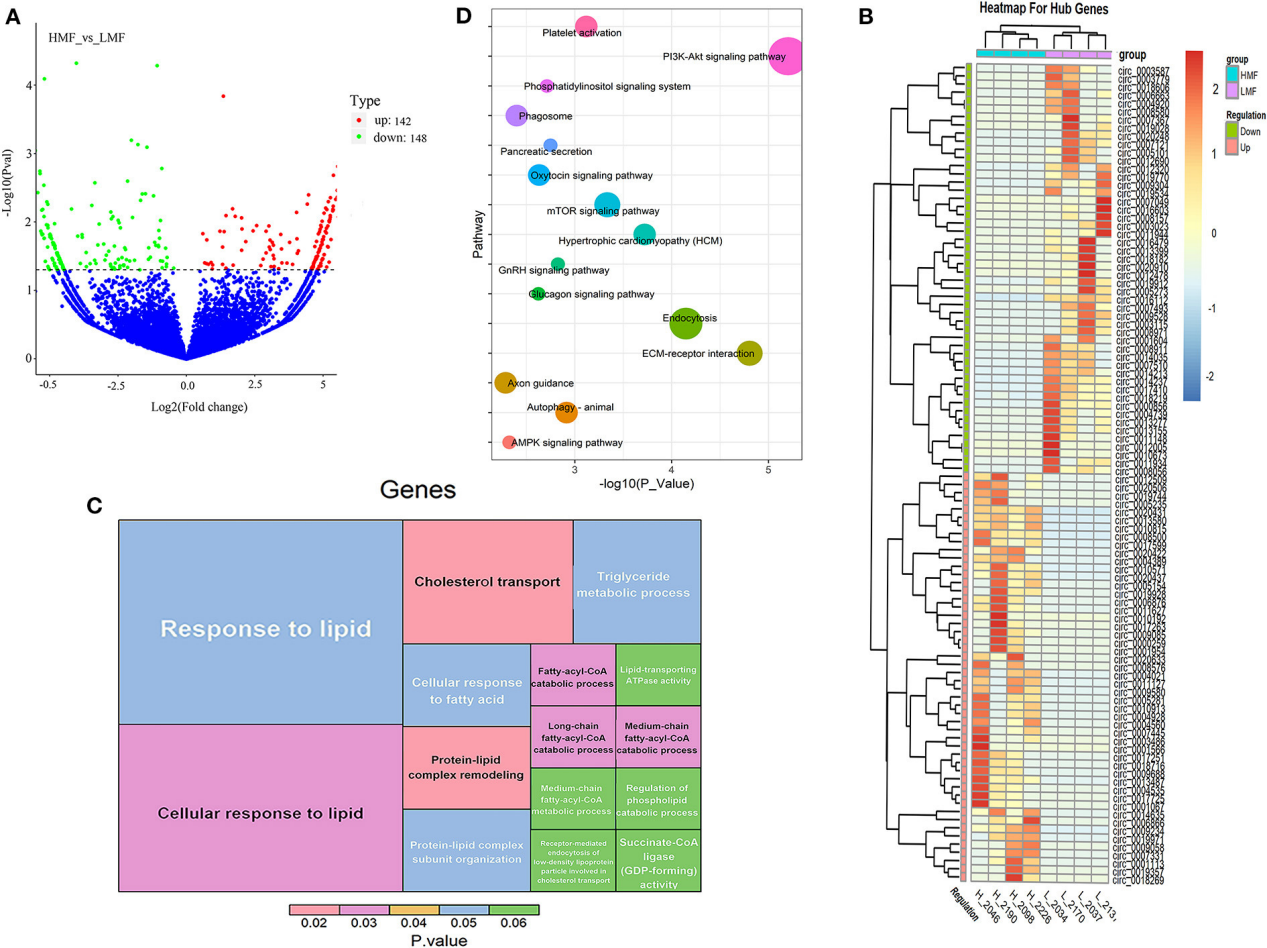
Expression and functional enrichment analysis of DE-circRNAs. **(A)** Volcano plots reveal the abundance of circRNAs in mammary epithelial cells of cows in the HMF and LMF groups. **(B)** Clustering of the 100 circRNA expression profiles with the most remarkable differences between the HMF and LMF groups. **(C)** Significantly enriched DE-circRNAs and lipid metabolism-related GO term (each lattice represents a term, the size of the lattice area represents how many genes are enriched for that term, and color represents significance). **(D)** DE-circRNAs were enriched in signaling pathways related to lipid metabolism (the size of the dots represents how many genes are enriched in that pathway).

A total of 290 DE-circRNAs were screened according to the fold-change and corrected *P*-value, of which 142 were significantly upregulated and 148 were significantly downregulated (Figure 2A). The 100 circRNAs with the most considerable fold-change and which differed most significantly between the HMF and LMF groups were selected and clustered according to their expression profiles, resulting in opposite expression patterns between the groups and similar clustering patterns of circRNA expression within groups (Figure 2B). Functional enrichment analysis of the source genes of DE-circRNAs revealed that 4,537 GO entries were enriched, containing 3,443 BPs, 445 CCs, and 649 MFs (Supplementary Table 5A). The significantly enriched GO entries involved in lipid metabolism were cholesterol transport, protein-lipid complex remodeling, cellular response to lipid, medium-chain fatty-acyl-CoA catabolic process, long-chain fatty-acyl-CoA catabolic process, and fatty-acyl-CoA catabolic process. The other GO entries that were not significant, but still related to lipid metabolism, were response to lipids, cellular response to fatty acid, medium-chain fatty-acyl-CoA metabolic process, and TAG metabolic process (Figure 2C). The source genes of DE-circRNAs were enriched in 226 KEGG signaling pathways, and 42 pathways were significantly enriched (Supplementary Table 5B), including the PI3K-Akt signaling, ECM-receptor interaction, endocytosis, and hypertrophic pathways. Fifteen signaling pathways may be closely related to lipid metabolism (Figure 2D). Considering that circRNAs can regulate the expression of their parental genes, we screened four circRNAs (circ_0007367, circ_0018269, circ_0015179, and circ_0001122) that may regulate milk fat metabolism through the enrichment analysis of parental genes. Their parental genes were significantly enriched in the GO entries of cellular response to lipid and response to lipid. In addition, the parental genes of circ_0015179 and circ_0007367 were found to be enriched in the PI3K-Akt signaling pathway and the mTOR signaling pathway, which are key signaling pathways related to lipid metabolism. The parental genes of circ_0018269 and circ_0001122 were found to be significantly enriched in the endocytosis and axon guidance signaling pathways, respectively. These results indicate that these circRNAs may have certain regulatory functions in lipid metabolism.

### Interaction network of candidate DE-circRNAs

**Figure 3:**
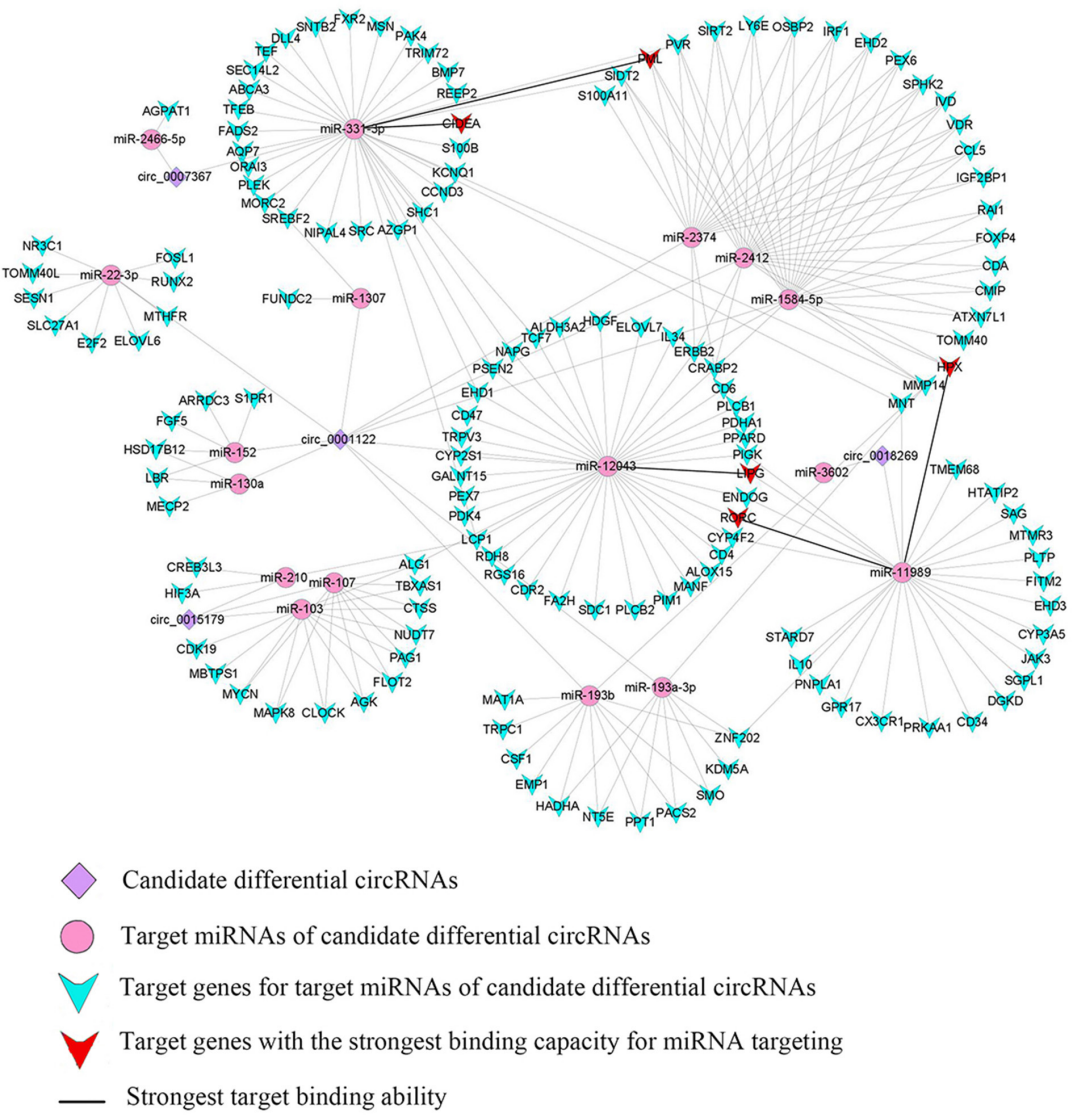
Interregulated network diagram of four candidate circRNAs.

**Figure 4:**
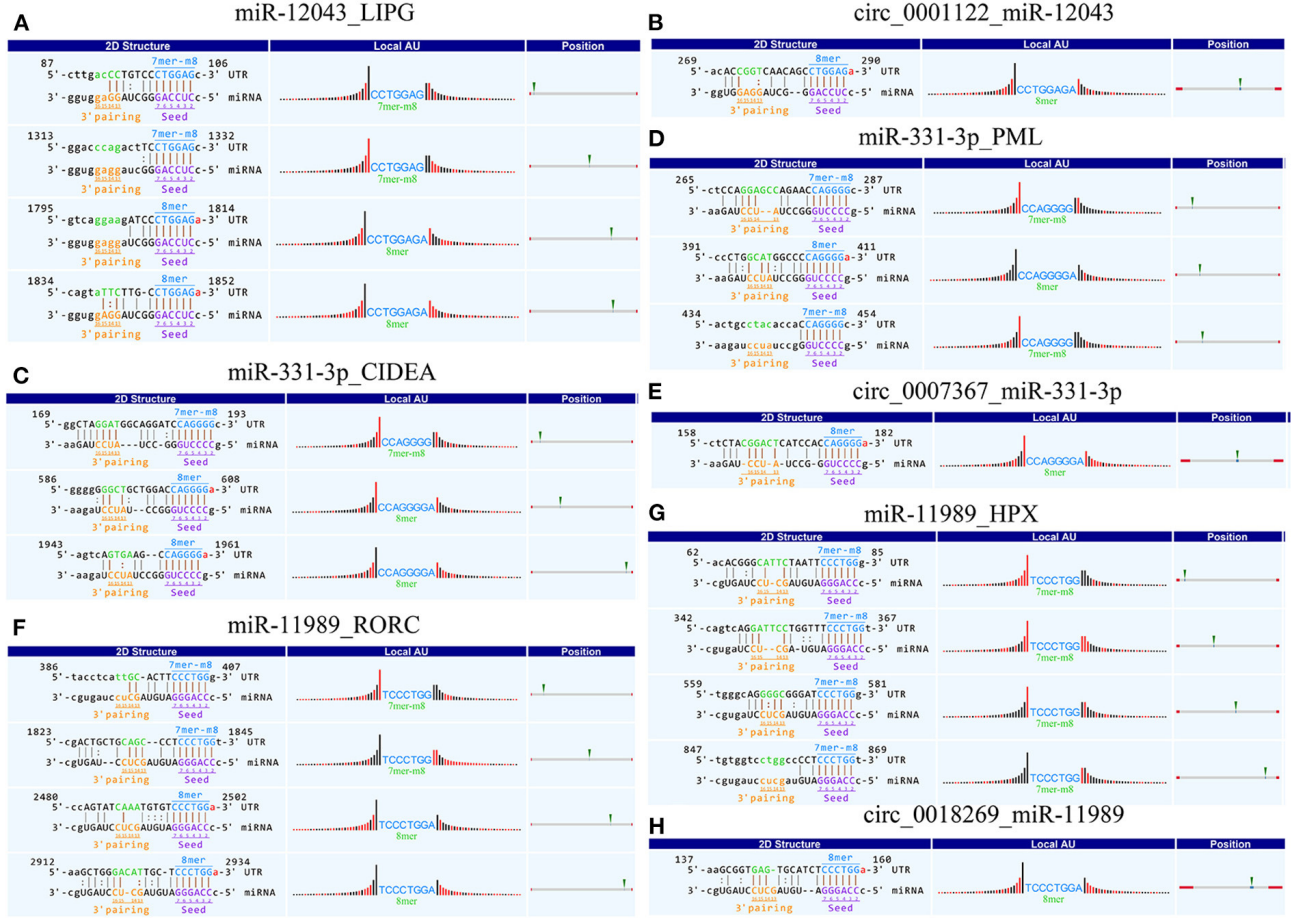
Schematic diagram of circRNA_miRNA_mRNA binding sites. **(A–H)** Targeted binding sites between circRNAs_miRNAs_mRNAs: pairing at bases 2–7 and 13–16 is particularly important for site recognition and is highlighted in brown. 8mer: 2–8 nucleotides matched perfectly, where 1 was A. 7mer-m8: 2–8 nucleotides matched perfectly, where 1 was not A. “|” denotes a perfect match. “:” denotes G:U pairing. “” denotes a mismatch. Local AU: Schematic diagram of AU weight on both sides of the seed site. This feature affects the accessibility of the site. The red bar indicates that the position is A:U. Position: The relative position of the site on the UTR, as close to both sides as possible.

Using Context+ ≤ −0.20 in TargetScan software and Free Energy ≤ −20 in miRanda software as criteria, potential miRNAs regulated by lipid metabolism-related candidate DE-circRNAs were screened. Based on the ceRNA theory, TargetScan and miRanda software were used to predict the target genes of miRNAs. High-scoring target genes were screened according to the criteria of Context+ ≤ −0.30 and Free Energy ≤ −30. Finally, their circRNA_miRNA_mRNA interaction regulatory network was constructed (Figure 3; Supplementary Table 6). According to the regulatory network diagram, there are 243 ceRNA regulatory networks involved in four DE-circRNAs (17 miRNAs and 146 genes). The circ_0001122 regulatory network contained the most miRNAs, of which miR-12043 had the most predicted target genes. Lipid metabolism-related genes, such as *LIPG, RORC, TCF7, PLCB1, ELOVL7, PLCB2, LCP1*, and *PPARD*, were all predicted as new targets of miR-12043. Among them, *LIPG* had a strong target binding ability with miR-12043; four target binding sites were predicted, and the best site among the four target binding sites was between nucleotides 87–106, which not only matched perfectly at nucleotides 2–8 but also at bases 13 and 14 (Figure 4A). The circ_0001122:miR-12043:LIPG interplay regulation network is one of the networks to be verified in our subsequent experiments. In this network, the circ_0001122:miR-12043 binding site was located between nucleotides 269–290 of circRNA (Figure 4B), with nucleotides 2–8 at the seed position matching exactly, and a total of 12 seed region sequences complementarily paired with the bases of the target gene. miR-2466-5p and miR-331-3p are the only two target miRNAs of circ_0007367, where miR-2466-5p is predicted to have only one target site with *AGPAT1*. Among the many target genes of miR-331-3p, the targeted binding ability with the *CIDEA* gene was the strongest, with a Context+ ≤ −0.99 and Free Energy ≤ −77.84. miR-331-3p had three target binding sites with *CIDEA* (Figure 4C), and the best binding site was the 169–193 nt sequence of *CIDEA*, with bases 2–8 and 13–16 fully paired. Secondly, strong interaction and regulation relationships were observed between miR-331-3p and *PML*, and three binding sites were also predicted (Figure 4D), with the optimal binding site being at nucleotides 265–287 of the target gene. The reciprocal sites of miR-331-3p and circ_0007367 are shown in Figure 4E, and the regulatory network they constitute is a potential network for probing the mechanism of milk fat regulation. Within the regulatory network relationship of circ_0018269_miR-11989_mRNA, there is a solid reciprocal regulatory relationship with *RORC* and *HPX*, constituting circ_0018269:miR-11989:RORC/HPX as a regulatory network for subsequent focus (their binding sites are shown in Figures 4F–H). miR-11989 was predicted to have four target binding sites with both *RORC* and *HPX*. The optimal targeting sites were at nucleotides 386–407 of *RORC* and 62–85 of *HPX*, respectively. circ_00015179 had a high binding ability to miR-210, miR-103, and miR-107, and the regulation of lipid metabolism by these miRNAs has been confirmed (Figure 3) (30, 31).

### Experimental validation of circRNA

**Figure 5:**
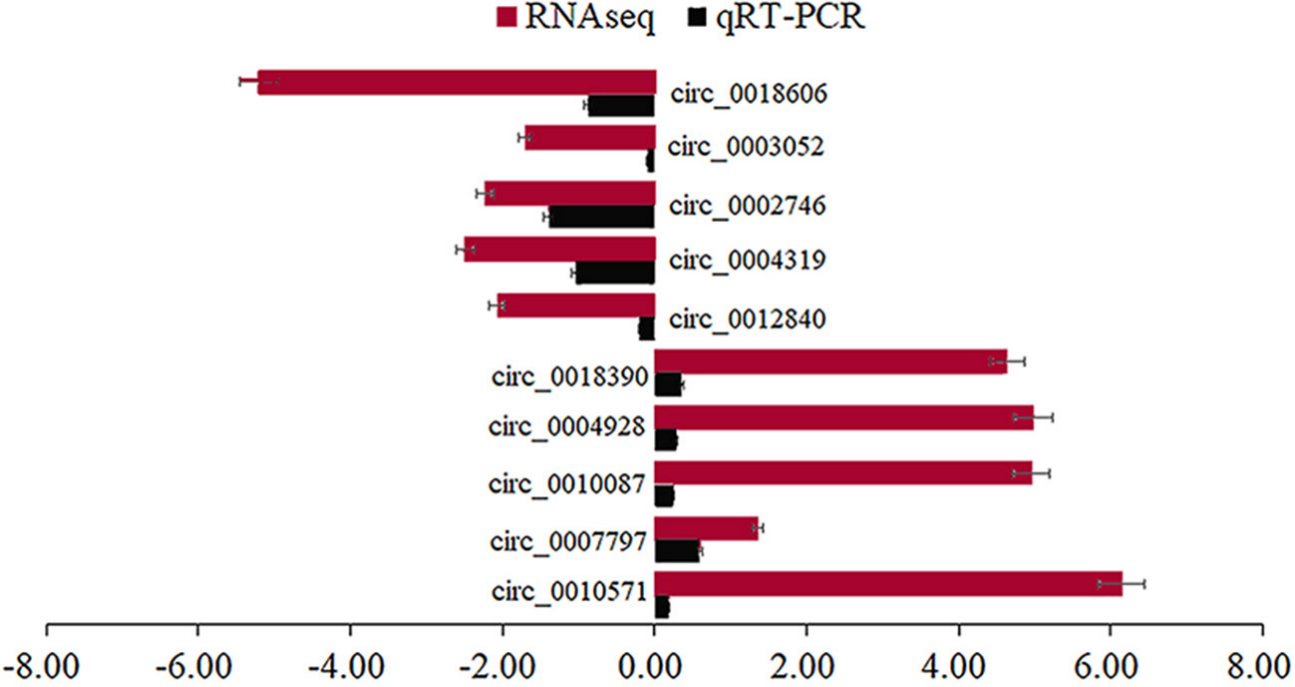
Different circRNAs verified using RT-qPCR.

**Figure 6:**
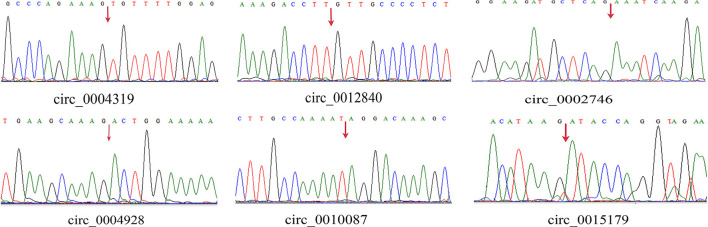
Head-to-tail splice junctions for the circRNAs were confirmed by DNA sequencing and are marked with a red arrow on the DNA sequence chromatograms.

**Figure 7:**
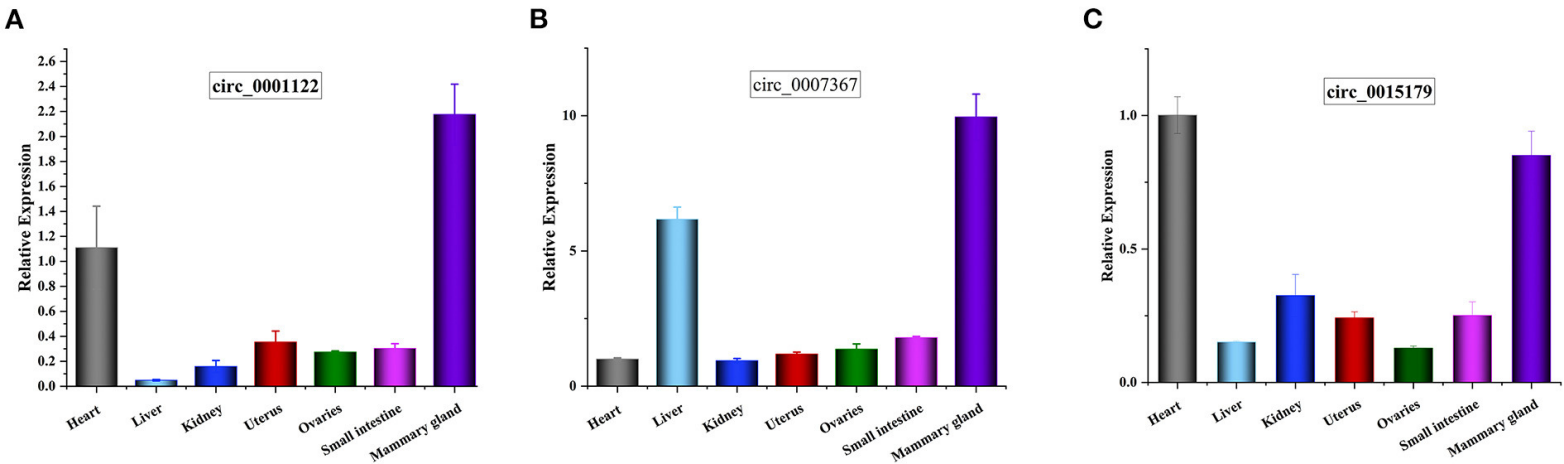
**(A–C)** Tissue expression profiles for circ_0001122, circ_0007367, and circ_0015179, respectively, horizontal axis shows different tissues, vertical axis shows relative expression levels.

We randomly selected five upregulated and five downregulated DE-circRNAs and analyzed their relative expression levels in the HMF and LMF groups of dairy cows using RT-qPCR. This result is consistent with the RNA-seq data (Figure 5), confirming the specific expression of these circRNAs in the HMF and LMF groups and validating the reliability of the transcriptome data. Then, six circRNAs were randomly selected and their reverse splicing sites were verified by PCR amplification with specific divergent primers and Sanger sequencing (Figure 6). To further demonstrate that the circRNAs screened in this study play an important function in milk lipid metabolism, the three circRNAs were selected for tissue expression analysis, and all of them were found to be expressed at relatively high levels in mammary gland tissue (Figure 7).

## Discussion

With the rapid development of high-throughput sequencing technology and bioinformatics, researchers have found that circRNAs are prevalent in various species and have essential physiological functions (32). Previous studies suggested that certain circRNAs may play critical roles in BMECs (33, 34). Wang et al. (35) studied and constructed a regulatory network of circRNA_microRNA_CD36 in the mammary gland tissue of heat-stressed cows. Similarly, Chen et al. (36) discovered that circ11103 and its circ11103:miR-128:PPARGC1A network regulate milk fat metabolism and fatty acid synthesis in cows. In another study, circRNAs derived from the *CSN1S1* gene were found to potentially act as a sponge for miR-2284 to regulate casein translation (37). In conclusion, circRNAs may regulate milk fat metabolism by regulating the expression of essential genes.

### Identification of key candidate circRNAs for milk fat metabolism

In this study, we identified abundant circRNAs and revealed the expression profile of circRNAs in BMECs in an attempt to further understand the molecular mechanism of milk lipid metabolism from the perspective of circRNAs. Based on the sequencing results, 290 DE-circRNAs were screened, and the reliability of the sequencing data was demonstrated by RT-qPCR and Sanger sequencing. As there is increasing evidence that circRNAs can regulate the expression of their parental genes (38), we performed GO and KEGG enrichment analysis on the host genes of DE-circRNAs to evaluate the function of circRNAs in BMECs.

The host genes of DE-circRNAs were enriched with related functions in lipid metabolism. The most significantly enriched GO term was cholesterol transport, suggesting that DE-circRNAs are most likely to play a regulatory function in the BP of cholesterol transport. Changes in intracellular cholesterol profoundly affect cellular functions, including proliferation, viability, signal transduction, membrane plasticity, and membrane migration (39, 40). The main functional pathways of DE-circRNAs were related to signaling and metabolic processes. The pathway with the most enriched genes and the most significant pathway was the PI3K-Akt signaling pathway, followed by the ECM-receptor interaction and the endocytosis pathway. The PI3K-Akt signaling pathway is necessary for mammary gland development and milk protein synthesis (41). The PI3K-Akt signaling pathway regulates multiple cellular activities, including cell growth, proliferation, survival, and metabolism (42). The interaction between the extracellular matrix (ECM) and mammary epithelial cells is essential for regulating cell proliferation, polarity, and apoptosis (43–45). Endocytosis is critical for regulating plasma membrane protein and lipid homeostasis, and endocytosis defects may lead to an irregular accumulation of proteins on the cytoplasmic membrane and lipids (46). BMECs control the uptake of basolateral blood molecules through endocytosis, affecting milk quality and quantity (47). In addition to the above signaling pathways, the more typical lipid metabolism signaling pathways mTOR and AMPK were enriched; the mTOR signaling pathway was associated with cell proliferation and the regulation of lipid and amino acid metabolism in mammals (48). The AMPK signaling pathway acts as an energy sensor that regulates metabolism in the body and cells, including lipid metabolism (49). Finally, four candidate DE-circRNAs (circ_0007367, circ_0018269, circ_0015179, and circ_0001122) were screened through the enrichment analysis from the above DE-circRNAs, suggesting that these DE-circRNAs may have potential regulatory mechanisms for mammary gland development and milk fat metabolism.

### ceRNA network construction of key candidate circRNAs

As a highly competitive endogenous RNA, circRNA is rich in miRNA-binding sites and can adsorb mRNAs through the “miRNA sponge” effect. Thereafter, it removes the inhibition of targeted mRNAs, and thus indirectly regulates gene expression. This phenomenon suggests that circRNAs can act as ceRNAs, and both circRNAs and mRNAs can target miRNAs. In this study, we focused on analyzing the DE-circRNAs screened by functional enrichment analysis and predicted the regulatory functions of these DE-circRNAs through the circRNA-miRNA-mRNA interaction regulatory network.

Among the four key candidate circRNAs screened by enrichment analysis, circ_0001122 can adsorb miR-22-3p to regulate *ELOVL6* and *SLC27A1* expression, which is consistent with Li et al. (50) prediction that miR-22-3p targets some genes related to lipid metabolism, including *ELOVL6*. Among the two targeted miRNAs of circ_0007367, miR-2466-5p only targeted *AGPAT1*, which plays a vital role in the lipid biosynthesis pathway (51), where the overexpression of *AGPAT1* gene increases fatty acid uptake, triacylglycerol production, and accumulation of fat droplets in cells (52). miR-103 and miR-107, which are hosted by pantothenate kinase genes, were targeted by circ_0015179 and were proposed to regulate cellular lipid metabolism (31). Their common target gene *CLOCK* can promote the expression of genes involved in lipid synthesis and glucose metabolism (53). To further identify the critical ceRNA networks regulating milk fat metabolism, we screened circ_0001122:miR-12043:LIPG, circ_0018269:miR-11989:RORC/HPX, and circ_0007367:miR-331-3p:CIDEA/PML through the strongest targeted binding ability of miRNA-mRNA as the critical ceRNA network to explore the mechanism underlying the regulation of milk fat. Lipase G (*LIPG*), a phospholipase located in the cytoplasm and cell membrane, has been shown to hydrolyze extracellular phospholipids from high-density lipoproteins, which are subsequently incorporated into intracellular lipids, and thus provide lipid precursors for cell metabolism (54, 55). *RORC* is associated with hepatic lipid and fatty acid metabolism and circadian pathways (56). *HPX* is associated with TAGs, and disruption of its expression impairs adipocyte differentiation (57). *CIDEA*, a lipid droplet (LD)-associated protein, protects LD from lipases, is highly expressed in brown adipose tissue, and regulates LD size and lipid storage (58, 59). *PML* is a crucial regulator of nuclear signaling events. Nuclear lipid droplets (nLDs) that retain *PML* are called lipid-associated *PML* structures (LAPS). Both nLDs and LAPS have lipid biosynthesis enzymes on their surfaces, which are active sites for nucleophospholipid and triacylglycerol synthesis and global lipid regulation (60). Tissue expression analysis revealed that circ_0001122, circ_0007367, and circ_0015179 all had relatively high levels of expression in breast tissue. These results provide strong evidence that the candidate DE-circRNAs play an important role in regulating milk lipid metabolism.

## Conclusions

In this study, 290 novel DE-circRNAs were identified in BMECs, greatly expanding the gene pool of bovine circRNAs. Among these, four DE-circRNAs related to lipid metabolism were screened by functional enrichment analysis, and five critical ceRNA interaction regulatory networks related to milk fat metabolism were screened from the ceRNA network constructed using these four DE-circRNAs. These ceRNAs are likely to be involved in the lactation process and provide novel insights into the mechanisms underlying milk fat metabolism.

## Data availability statement

The datasets presented in this study can be found in online repositories. The names of the repository/repositories and accession number(s) can be found in the article/Supplementary material.

## Ethics statement

Ethical review and approval was not required for the animal study because the sample used in this study was milk, which is obtained from Holstein cows, the main large milk producing animal, in the normal production process. Written informed consent was obtained from the owners for the participation of their animals in this study.

## Author contributions

XF was mainly responsible for data analysis and manuscript writing. ZC provided reference suggestions for data visualization. TM performed the isolation and culture of mammary epithelial cells. YG and JZ revised the manuscript and provided reagents. BY, YW, RM, and JL conducted formal analysis. All authors were involved in sample collection, manuscript conception, and experimental design.

## Funding

This project is supported by the special breeding project of high-quality and high-yield dairy cows in the Ningxia Autonomous Region (Grant No: 2019NYYZ05).

## Conflict of interest

The authors declare that the research was conducted in the absence of any commercial or financial relationships that could be construed as a potential conflict of interest.

## Publisher's note

All claims expressed in this article are solely those of the authors and do not necessarily represent those of their affiliated organizations, or those of the publisher, the editors and the reviewers. Any product that may be evaluated in this article, or claim that may be made by its manufacturer, is not guaranteed or endorsed by the publisher.
