# Supplementary material for: CircRNA screening and ceRNA network construction for milk fat metabolism in dairy cows
Source: Front Vet Sci. 2022 Nov 10;9:995629. doi: 10.3389/fvets.2022.995629 (PMC9684208; doi:10.3389/fvets.2022.995629)
Supplement: Supplementary file 1 [file Table_1.DOCX]

**Supplementaty table 1 Ingredient and nutrient composition in diet (dry matter basis) %**

| Ingredient | Content | Composition | Content |
| --- | --- | --- | --- |
| Alfalfa | 16.22 | Dry matter (kg) | 17.28 |
| Corn silage | 51.32 | Net energy for lactating cow (MJ/kg) | 7.76 |
| Tablet corn | 10.82 | Crude protein | 18.31 |
| Soybean meal | 10.82 | Neutral detergent fiber | 35.84 |
| Cotton meal | 5.41 | Acid detergent fiber | 21.85 |
| 10 % premix | 5.41 | Fat | 2.59 |
|  |  | Ca | 0.52 |
| Total | 100 | P | 0.35 |

Note: Each kg of premix contains 800 000 IU of V_A_, 200 000 IU of V_D_, 4 000 mg of V_E_, 1 200 mg of Cu, 6 000 mg of Fe, 4 000 mg of Mn, 4 000 mg of Zn, 40 mg of I, 40 mg of Co and 32 mg of Se.
