# Supplementary material for: CircRNA screening and ceRNA network construction for milk fat metabolism in dairy cows
Source: Front Vet Sci. 2022 Nov 10;9:995629. doi: 10.3389/fvets.2022.995629 (PMC9684208; doi:10.3389/fvets.2022.995629)
Supplement: Supplementary file 2 [file Table_2.DOCX]

| circRNA | Primer sequences(5'→3') | Product length/bp | Annealing temperature/℃ |
| --- | --- | --- | --- |
| circ_0010571 | F: AGTCGATTCCCTTCATGCT | 117 | 53.0 |
|  | R: TAGGTATTCTCGCCGTTTA |  | 50.8 |
| circ_0007797 | F: TGGCATCTTTGCTGCACACGCT | 136 | 59.5 |
|  | R: TGCTCTGCTCAGGGACTCATTG |  | 59.5 |
| circ_0010087 | F: GGAAGATGTGCAGGAGATGC | 106 | 57.5 |
|  | R: AGGTGGGACACTCTTTCTGG |  | 57.5 |
| circ_0004928 | F:AAAGAGAGCAGGAGCTGACA | 143 | 55.4 |
|  | R:TTGGCTAACAAGCAGCACTC |  | 55.4 |
| circ_0018390 | F:GCAAGCCGGTTTGACATCT | 133 | 55.2 |
|  | R:CTTCCTGCTCCTCTGGGAAA |  | 57.5 |
| circ_0012840 | F: TTGTGCGGTGCTGGTGAAGAC | 117 | 59.5 |
|  | R: TAGAGGCAGATGGACAGGGAG |  | 59.5 |
| circ_0004319 | F: GGTGTTGATGCTTTGGGT | 166 | 52.6 |
|  | R: GGGCCTTCATTGTCTGTT |  | 52.6 |
| circ_0002746 | F: ATACTAAGCGGACCAGAACCT | 123 | 55.6 |
|  | R: AGAGGACGGGCTGATTAAAGT |  | 55.6 |
| circ_0003052 | F: GGCCCATTGGTGTTACTA | 100 | 52.6 |
|  | R: CGCTGTGTCTCATCGTTT |  | 52.6 |
| circ_0018606 | F:AACGGCTATTCAACCCTGGA | 152 | 55.4 |
|  | R:TAGGCAGAGTTTGGGAGGTG |  | 55.7 |
| circ_0015179 | F: GTTCTGTTGCTACTCTCCT | 84 | 45.6 |
|  | R:TCACCTGGTATCTTATGTC |  | 44.0 |
| circ_0001122 | F:GTGTACCACTACCGCATCAA | 115 | 53.9 |
|  | R:TCCTTAGAGTTCCAACGAGC |  | 54.6 |
| circ_0007367 | F:ATACTAAGCGGACCAGAACCT | 123 | 55.3 |
|  | R:AGAGGACGGGCTGATTAAAGT |  | 55.7 |

**Supplementary table 2 RT-qPCR and PCR primers for DE-circRNAs**
